# Enrichment of lung microbiome with supraglottic taxa is associated with increased pulmonary inflammation

**DOI:** 10.1186/2049-2618-1-19

**Published:** 2013-07-01

**Authors:** Leopoldo N Segal, Alexander V Alekseyenko, Jose C Clemente, Rohan Kulkarni, Benjamin Wu, Hao Chen, Kenneth I Berger, Roberta M Goldring, William N Rom, Martin J Blaser, Michael D Weiden

**Affiliations:** 1 André Cournand Pulmonary Research Laboratory, Bellevue Hospital Center/New York University School of Medicine, New York, NY, USA; 2 Division of Pulmonary and Critical Care Medicine, New York University School of Medicine, New York, NY, USA; 3 Department of Medicine, New York University School of Medicine, 462 First Ave 7 W54, New York 10016, NY, USA; 4 Center for Health Informatics and Bioinformatics, New York University School of Medicine, New York, NY, USA; 5 Division of Genetics and Genomic Sciences, Mount Sinai School of Medicine, New York, NY, USA

**Keywords:** Lung, Bronchoscopy, Microbiome, Inflammation

## Abstract

**Background:**

The lung microbiome of healthy individuals frequently harbors oral organisms. Despite evidence that microaspiration is commonly associated with smoking-related lung diseases, the effects of lung microbiome enrichment with upper airway taxa on inflammation has not been studied. We hypothesize that the presence of oral microorganisms in the lung microbiome is associated with enhanced pulmonary inflammation. To test this, we sampled bronchoalveolar lavage (BAL) from the lower airways of 29 asymptomatic subjects (nine never-smokers, 14 former-smokers, and six current-smokers). We quantified, amplified, and sequenced 16S rRNA genes from BAL samples by qPCR and 454 sequencing. Pulmonary inflammation was assessed by exhaled nitric oxide (eNO), BAL lymphocytes, and neutrophils.

**Results:**

BAL had lower total 16S than supraglottic samples and higher than saline background. Bacterial communities in the lower airway clustered in two distinct groups that we designated as pneumotypes. The rRNA gene concentration and microbial community of the first pneumotype was similar to that of the saline background. The second pneumotype had higher rRNA gene concentration and higher relative abundance of supraglottic-characteristic taxa (SCT), such as *Veillonella* and *Prevotella*, and we called it pneumotype_SCT_. Smoking had no effect on pneumotype allocation, α, or β diversity. Pneumotype_SCT_ was associated with higher BAL lymphocyte-count (*P*= 0.007), BAL neutrophil-count (*P*= 0.034), and eNO (*P*= 0.022).

**Conclusion:**

A pneumotype with high relative abundance of supraglottic-characteristic taxa is associated with enhanced subclinical lung inflammation.

## Background

The gut microbiome modulates host mucosal immune response [1,2]. In contrast, despite emerging data on airway microbiota, little is known about the role of the lung microbiome in modulating pulmonary mucosal immune response. Along the human airways, structures above the vocal cords are exposed to high bacterial burden producing contamination of lower airway samples with oropharyngeal secretions [3,4]. Classical culture techniques fail to fully describe microbial communities of the lower airways due to difficulties in growing fastidious bacteria, thus identification using marker gene sequencing is a more promising approach [5-11]. Such studies identified bacterial rRNA genes of oral cavity anaerobes such as *Prevotella* and *Veillonella* in the lower airways of normal individuals [6,8,12-16].

**Table 1:** Demographic, pulmonary functional, and BAL cell differential in 29 participants

Age (years)	62 (55–66)
Male	72%
Caucasian	82%
BMI	27 (24–30)
Smoking status	
Current	21%
Former	48%
Never	31%
Pack/year (Smokers)	43 (30–50)
PFT	
*Spirometry*^a^	
FVC (% Predicted)	97 (85–104)
FEV_1_ (% Predicted)	94 (86–104)
FEV_1_/VC	73.4 (70.0-83.4)
*Lung volumes*	
TLC (% Predicted)	102 (92–114)
FRC (% Predicted)	90 (94–107)
RV/TLC	0.32 (0.25-0.38)
*DLCO (% Predicted)*	91 (80–105)
*IOS (R*_*5*_*cmH*_*2*_*O/L/s)*	3.0 (2.5-3.4)
*eNO (ppb)*	23.2 (10.9-34.2)
BAL cellsx10^3^/mL BALF	
Macrophages	164.0 (138.2-249.7)
Lymphocytes	15.6 (8.3-26.6)
Neutrophils	3.4 (2.6-5.6)
Eosinophils	0.0 (0.0-1.6)

Data presented as % or Median (IQR).

^a^Data based on NHANES predicted values.

Microaspiration of small volumes of oropharyngeal secretions occurs in healthy people [17]. The risk for microaspiration is increased in smoking-related lung disease due to reduced coordination of breathing with swallowing and gastro-esophageal reflux [18,19]. Both microaspiration and impaired mechanical clearance in smokers may lead to increased lower airway colonization with oral microbiota. Prior studies suggest that in moderate to severe chronic obstructive pulmonary disease (COPD), the lung microbiome is enriched with bacteria or bacterial products common to the oral cavity [7,9,20,21]. However, these studies have focused on advanced COPD patients, in whom frequent antibiotic and corticosteroid use may affect the bacterial communities of the lower airways. Studies of early disease and asymptomatic cases would avoid these potential confounding effects.

Increased toll-like receptor signaling has been associated with pulmonary inflammation in advanced COPD, offering a mechanisms by which microbial inhabitants in the lung might be relevant for the development of smoking-related lung injury [22,23]. Furthermore, randomized clinical trials provide indications that antibiotics, especially macrolides, may reduce COPD exacerbations [24,25]. However, it is unclear whether the beneficial effect is due to the antibiotic or to the anti-inflammatory properties of these drugs. An in-depth understanding of the lung microbiome and its association with mucosal inflammatory response is needed to understand potential mechanisms of lung mucosal immune regulation. Here, we hypothesized that the enrichment of the lung microbiome of asymptomatic subjects with supraglottic-characteristic taxa is associated with lung inflammation. To avoid potential confounders, we selected asymptomatic smokers and never-smokers with preserved lung function and no recent exposure to antibiotics or immune modulators, to assess lung microbiome characteristics.

## Methods

### Participants

We enrolled 29 asymptomatic subjects(nine never-smokers, 14 former-smokers, and six current-smokers) for research bronchoscopy. All participants signed informed consent to take part in this study and the research protocol was approved by the human subjects review committees of New York University and by Bellevue Hospital Center (New York, NY) institutional review boards. For all participants, exclusion criteria were: FEV_1_<70%; recent treatment with antibiotics or steroids in the prior 3 months; cardiovascular disease (abnormal EKG, known or suspected coronary artery disease, or congestive heart failure); diabetes mellitus; renal or liver disease; lung cancer; heavy alcohol use (>6 beers daily). During the week prior to bronchoscopy, all participants underwent research pulmonary function testing for physiologic phenotyping, which included spirometry, lung volumes, diffusion, impulse oscillometry (IOS), and exhaled nitric oxide (eNO) as a non-invasive measure of airway inflammation [26]. In eight participants, eNO could not be reliably measured due to high levels of ambient NO that was associated with inability to identify a clear plateau on the exhaled NO *vs*. time tracing.

### Bronchoalveolar lavage

For all 29 participants, we used a nasal approach to perform research bronchoscopy. Since we previously observed low bacterial loads in nasal brushing [27], we used an optimized nasal bronchoscopy in an attempt to minimize the potential for contamination of the bronchoscope channel with upper airway microbiota. Given the concern for potential carry-over of upper airway microbiome to the lower airways, we avoid suctioning until the scope was in position for sampling. If during bronchoscopy, visualization was obscured, we instilled saline through the bronchoscope channel to clear the optics. In a subgroup of 15 participants, we used two bronchoscopes to evaluate potential carry-over. In this subgroup, we obtained a background sample from sterile saline and sterile normal saline passed through the bronchoscope channel. We then passed the first bronchoscope until vocal cords were visualized to obtain a supraglottic sample. The second bronchoscope was passed to obtain BAL in two different segments. The initial BAL was from a segment of the left lung (lingula) and the second BAL was from a segment of the right lung (right middle lobe, Additional file 1: Figure S1). We used three aliquots of 50 mL of saline for each BAL. On average, 50% of the instilled volume was recovered. The BAL samples were immediately placed on ice and processed within 30 min of acquisition. Total cell count and BAL cell differentials (500 cells counted) were performed to assess counts of macrophages, lymphocytes, and neutrophils. BAL fluid aliquots were frozen at −80°C for DNA isolation.

### Bacterial DNA quantification and 454 pyrosequencing

DNA was isolated from acellular BAL fluid after centrifugation at 500 g for 10 min at 4°C. Three methods were used to ensure uniform bacterial lysis: a freeze-thaw cycle, lysozyme, and a 56°C heat step at the beginning of the DNA isolation process. DNA was then extracted with an ion exchange column (Qiagen). Total bacterial and human DNA levels were determined by quantitative PCR (qPCR). We performed high-throughput sequencing of the bacterial 16S rRNA gene using the Roche 454 amplicon sequencing protocol. Each sample was individually barcoded using a unique 12nt sequence during amplification of the V1-V2 regions of the 16S gene [28]. Further details described in Additional file 2 on line supplement.

### Upstream informatics analysis of the 16S sequences

The obtained 16S rRNA sequences were analyzed using the Quantitative Insights into Microbial Ecology (QIIME) pipeline for analysis of community sequence data [29]. Processing consisted of the following steps: (1) demultiplexing and filtering of short (<150 nt) and low quality reads; (2) *de novo* clustering of the sequences into operational taxonomic units (OTUs) with the UCLUST program using a 97% similarity threshold [30]; (3) taxonomical assignment of each OTU by running the RDP Classifier [31] at 80% bootstrap confidence on a selected representative sequence from each OTU; (4) alignment of representative sequences using PyNAST [32] with the Greengenes core-set alignment template; (5) phylogenetic tree reconstruction from the representative sequences for each OTUs using the FASSTTREE program [33]; and (6) UniFrac distance calculations [34]. For each sample, the proportion of reads at the OTU or genus levels was used as a measure of the relative abundance of each type of bacteria in a specimen. The exact commands run are available in the Supplement.

### Univariate analysis of association between microbiome and immunological phenotype

The absolute OTU sequence counts were normalized to obtain the relative abundances of the microbiota in each sample. These relative abundances at 97% OTU similarity and each of the five higher taxonomic levels (phylum, class, order, family, genus) were tested for univariate associations with clinical variables. To decrease the number of features, we only focused on major taxa and OTUs, defined as those having mean relative abundance >1% in at least one sample.

Since the distributions of microbiome data are non-normal, and no distribution-specific tests are available, we used non-parametric tests of association. For association with discrete factors, we used either the Mann–Whitney test (in the case of two categories) or the Kruskal Wallis ANOVA (in case of >2 categories). For tests of association with continuous variables, we used non-parametric Spearman correlation tests. False discovery rate (FDR) was used to control for multiple testing [35]. Weighted UniFrac was used to measure β diversity of bacterial communities and to perform principal coordinate analysis (PCoA) [36]. We used the ade4 package in R to PCoA on weighted Unifrac distances [37]. To avoid negative eigenvalues in the analysis, we used the Cailliez method to convert the weighted Unifrac distance matrix into a closest corresponding matrix with Euclidean properties, which was further used for PCoA [38]. Hierarchical clustering on the relative abundance profiles was used to establish deep branching, which was interpreted as evidence for distinct pneumotypes.

### Classification of streptococcus OTUs

For classification of Streptococcus OTUs, 16S rRNA sequences were aligned to the Greengenes database using online Blast tool (http://greengenes.lbl.gov/cgi-bin/nph-blast_interface.cgi). Top hits were retained. A perfect match was used for final classification if found, otherwise top hit is reported. In case two species we found more than once, both are reported.

## Results

### Clinical phenotype

Table 1 shows demographics, clinical characteristics, pulmonary function, and BAL cell counts for the 29 participants of this cohort. Enrolled subjects had normal FEV_1_ (median (IQR) = 94(86–104)% predicted) and FVC (97(85–104)% predicted), and normal or mild obstruction as quantified by FEV_1_/VC (73.4 (70.0-83.4)). Total airway resistance as assessed by IOS also was in the normal range (R_5_ = 3.0(2.5-3.4) cmH_2_O/L/s). Diffusion was normal (91(80–105)% predicted). Exhaled NO ranged from normal to elevated (range, 6.9-46.3 ppb). BAL cell differentials were normal with major predominance of alveolar macrophages and no neutrophilia or eosinophilia. In total, these data show that our cohort of asymptomatic subjects had no major pulmonary function abnormalities or obvious clinical evidence of disease.

### Bacterial 16S rRNA gene qPCR and sequencing

**Figure 1:**
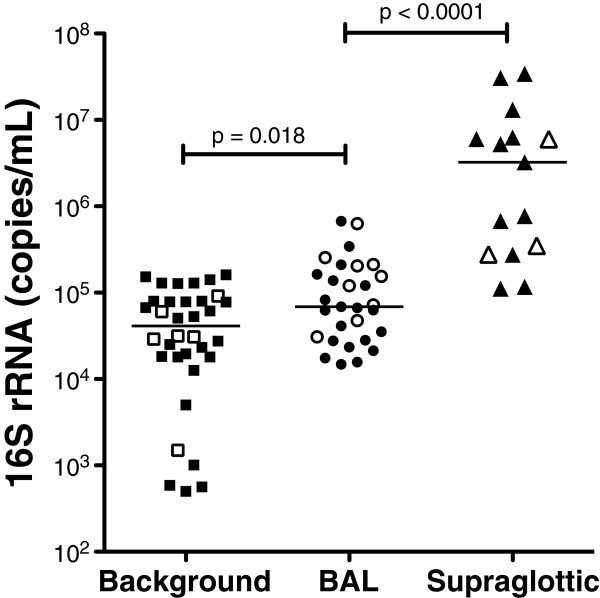
**Comparison of bacterial loads in background, BAL, and supraglotic samples.** Samples from background were obtained from either sterile saline (open square) or from sterile saline flushed through bronchoscope (closed square) and samples from BAL (open circles for never-smokers and closed circles for smokers) and supraglotic (open triangles for never-smokers and closed triangles for smokers) were obtained via bronchoscopy as described (see Additional file 1: Figure S1 for details). To detect bacterial load, universal primers for bacterial 16S rRNA were used in combination with a TaqMan Probe. Differences in bacterial loads were evaluated using Mann–Whitney U test.

We next examined bacterial load in the upper airways, lower airways, and background. Supraglottic samples had the highest 16S rRNA gene concentration (3,237,500 (276,625-6,262,500) copies/μL, Figure 1). 16S concentrations in BAL were approximately 50-fold lower (68,875 (29,475-183,312) copies/μL, *P*<0.001). Despite extensive overlap in bacterial load between background and BAL samples, background samples had lower 16S rRNA gene concentration (41,195 (18,035-79,875) copies/μL, *P*= 0.018). After trimming and quality control filtering, we obtained 641,847 rDNA sequences.

454 sequencing yield no significant difference in the number of reads per sample between rRNA obtained from background, BAL, and supraglottic specimens (median = 6,116, 5,532, and 7,961 high quality reads per sample, respectively, *P*= 0.12). The number of OTUs observed at 97% homology was significantly lower in background samples (93 (79–112)) compared with BAL (136 (98–158), *P*<0.001) and supraglottic samples (112 (102–140), *P*= 0.004).

**Figure 2:**
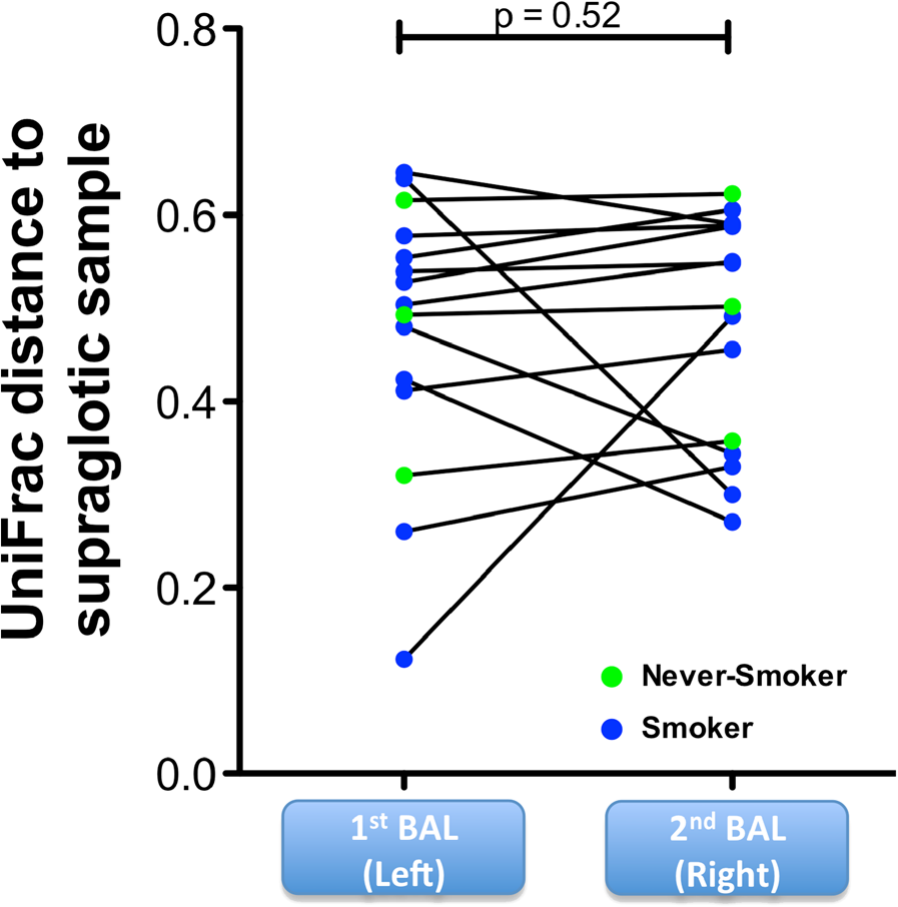
**Evaluation ofcarry-over of supraglottic microbiome compared with first and second BAL.** To evaluate carry-over of supraglotic-characteristic taxa to the lower airways, we evaluated the microbiome of the first and second BAL return in 15 cases where a separate bronchoscope was used to obtain a supraglotic sample (see Additional file 1: Figure S1 for details). Paired comparison of UniFrac distances between supraglottic and first BAL samples compared with supraglottic and second BAL samples (Wilcoxon rank-sum test).

**Figure 3:**
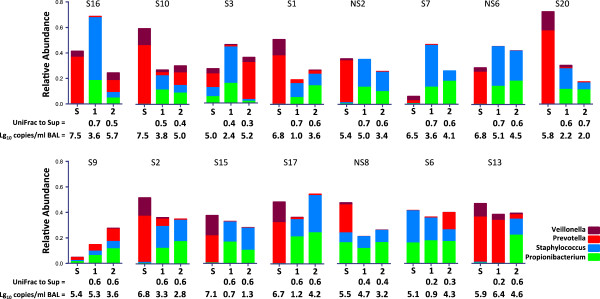
**Comparison of supraglottic bacterial communities with first and second BAL microbiome.** Relative abundances of *Veillonella*, *Prevotella*, *Propionibacterium*, and *Staphylococcus* in 15 subjects with paired supraglottic (S), first BAL (1), and second BAL (2). *Veillonella* and *Prevotella*are the two most abundant taxa in supraglottic samples while *Propionibacterium* and *Staphylococcus* are the two most abundant taxa in background. UniFrac distance to supraglottic and bacterial load adjusted per mL of BALF (log_10_ 16S qPCR) are shown below the bar graph of relative abundance. Overall, first BAL is not consistently closer to supraglottic than second BAL.

To ensure that research bronchoscopy did not systematically carry-over supraglottic secretions into the lower airway, we compared the matched sequencing samples from 15 subjects (three never-smokers and 12 smokers) from the supraglottic area, with the first BAL obtained from a segment in the lingula and the second BAL obtained from a right middle lobe segment. UniFrac distances were calculated between supraglottic and both first and second BAL (Figure 2). There were no significantly different UniFrac distances to supraglottic microbiome between first and second BAL, providing evidence that systematic carry-over did not occur. Analysis using unweighted UniFrac analysis yielded similar results. Figure 3 shows data from the 15 patients with complete supraglottic, first BAL, and second BAL data. The relative abundance of highly represented taxa in the supraglottic (*Prevotella* and *Veillonella*) and saline background (*Staphylococcus* and *Propionibacterium*) are shown in the bar graph with 16S rRNA copies/mL and Unifrac distance shown below each sample. There was no consistent ‘dilution’ of bacterial load and/or relative abundances of supraglottic taxa in subsequent BAL samples. As an example, in case S10, the first BAL had lower relative abundance of *Prevotella* and *Veillonella* and lower total bacterial load by qPCR (6,902 copies/mL) than the supraglottic sample (bacterial load = 34,250,000 copies/mL). Compared to first BAL, the second BAL had increased relative abundance of *Prevotella* and *Veillonella* (total bacterial load 103,300 copies/mL). Since the bronchoscope channel was not in contact with the upper airway between the first and second BAL, the increased relative abundance of *Prevotella*/*Veillonella* and total bacterial load between the first and second BAL in this example could not have been due to carry-over of supraglottic secretions contaminating the bronchoscope. From these observations, we conclude that enrichment of lower airway microbiome with supraglottic-characteristic taxa (SCT) most likely results from microaspiration, rather than from bronchoscopic carry-over.

### Distinct pneumotypes in the lung microbiome

**Figure 4:**
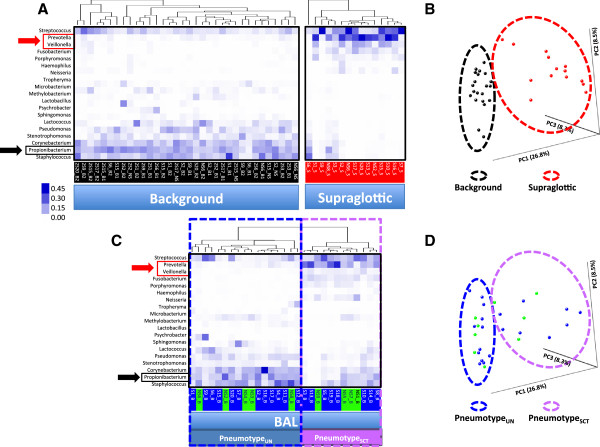
**Clustering analysis of background, supraglotic, and BAL microbiota patterns. (A)** Heat map of unsupervised hierarchical clustering of most abundant OTUs at a genus level (relative abundance ≥5% in any sample) in background and supraglottic samples. Background microbiome (sterile saline and saline through bronchoscope, see Additional file 1: Figure S1 for details) is enriched with *Staphylococcus*, *Propionibacterium*, and *Corynebacterium*, while the supraglottic microbiome is enriched with *Prevotella* and *Streptococcus*. **(B)** PCoA analysis based on weighted UniFrac distances clustered background samples separated from supraglotic samples. **(C)** Heat map of unsupervised hierarchical clustering of BAL samples. Never-smokers are indicated with green labels and smokers with blue labels. Dendrogram shows deep cleft that identified two major BAL microbiomes: one characterized by high relative abundance of *Staphylococcus*, *Propionibacterium*, and *Corynebacterium* which we called Pneumotype_UN_ and a second with high relative abundance of *Prevotella*, *Veillonella*, and *Streptococcus* (pneumotype_SCT_). **(D)** PCoA analysis based on weighted UniFrac distances differentiate the same BAL samples in the same two well-defined clusters (never-smokers in green dots and smokers in blue dots).

We next asked whether distinct microbiomes could be identified in the lower airways. We used unsupervised hierarchical clustering to assess the structure of the microbiome in the background, supraglottic, and BAL samples. Background sterile saline contained high relative abundances of *Propionibacterium*, *Staphylococcus*, *Corynebacterium*, *Stenotrophomonas*, and *Pseudomonas* (Figure 4, panel A). Supraglottic samples contained high relative abundances of *Prevotella*, *Veillonella*, *Streptococcus*, *Fusobacterium*, and *Porphyromonas*. PCoA of weighted UniFrac distances confirms that the supraglottic and background samples are distinct (panel B). Similar results were found when unweighted UniFrac was used (data not shown).

Unsupervised hierarchical clustering of BAL samples exhibited deep branches into two distinct clusters that we called ‘pneumotypes’ (Figure 4, panel C). The first pneumotype is similar to background samples with high relative abundance of *Propionibacterium* (panel C). This similarity to background microbiome makes it difficult to determine presence of unique lung taxa and therefore we labeled it undetermined pneumotype (pneumotype_UN_). This pneumotype was present in 12/20 (60%) smokers and 5/9 (55%) never-smokers. PCoA of weighted UniFrac distances shows that BAL samples characterized as pneumotype_UN_ were in similar spatial location as background samples (panels B and D, Additional file 3: Figure S2). Similar results were found when unweighted UniFrac was used.

The second pneumotype had high relative abundance of supraglottic-characteristic taxa (SCT), such as *Prevotella* and *Veillonella* (Figure 4, panels A and C). We named this group pneumotype_SCT_. PCoA of weighted UniFrac distances indicated that BAL samples characterized as pneumotype_SCT_ were in similar spatial locations as supraglottic samples (panels B and D, Additional file 3: Figure S2). Pneumotype_SCT_ had four-fold higher total 16S rRNA gene concentrations compared with pneumotype_UN_ (183,312 (72,343-320,442)*vs*. 41,300 (22,375-96,000) copies/mL, *P*= 0.003). The 16S concentration in pneumotype_UN_ was not significantly different from background.

Compared with the never-smoker group, the lung microbiome of smokers had similar distribution along PCoA as well as α and β diversity (Additional file 4: Figure S3). Smoking did not have significant impact on pneumotype allocation.

### Background microbiome

**Figure 5:**
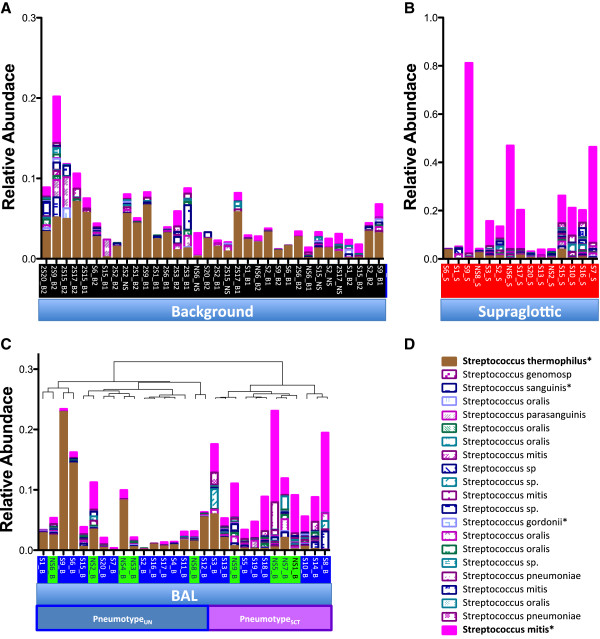
**Classification of *Streptococcus *OTUs in background, supraglottic, and BAL microbiota.** Twenty-one Streptococcus OTUs were aligned to the Greengenes database of 16S rRNA sequences. In each case, the top hit (>96%) was used for final classification. Perfect match was indicated (*) if found. **(A) **Background samples contained predominantly *Streptococcus thermophilus*. **(B)** Supraglottic samples contained predominantly *Streptococcus mitis*. **(C)** Relative abundances of all *Streptococcus* OTUs in all 29 BAL samples clustered as indicated in Figure 3 (never-smokers indicated with green label and smokers with blue label). Pneumotype_UN_ is enriched with *Streptococcus thermophilus* while Pneumotype_SCT_ has higher relative abundances of *Streptococcus mitis*.

Since 16S rRNA from the genus *Streptococcus* was found in background, BAL, and supraglottic samples, we evaluated the relative abundances of each *Streptococcus* OTU in all samples (Figure 5). Classification of *Streptococcus* OTU by Greengenes Blast showed that background samples were enriched with *Streptococcus thermophilus* (relative abundance 0.023 (0.010-0.036)), a known environmental contaminant [39]. Similarly, pneumotype_UN_ had higher relative abundance of *Streptococcus thermophilus* than pneumotype_SCT_ (0.014 (0.005-0.044)*vs*. 0.003 (0.002-0.017), *P*= 0.04). Conversely, supraglottic samples were enriched with *Streptococcus mitis* (relative abundance 0.0520 (range, 0.0084-0.1625)), a well-known member of the oral microbiota and an opportunistic pathogen. Pneumotype_SCT_ also was enriched with *Streptococcus mitis* (0.0057 (0.0024-0.0104)*vs*. 0.0441 (0.0266-0.0617), for pneumotype_UN_ and pneumotype_SCT_ respectively, *P*<0.0001).

The presence of bacterial DNA in the sterile saline used to perform the BAL led to the expected presence of background in each BAL sample. We therefore tested whether removal of sequences commonly found in the saline altered pneumotype definition. Subtraction of these ‘saline OTUs’ (≥1% relative abundance in saline) from BAL samples did not alter sample allocation in the unsupervised hierarchical clustering (data not shown). However, subtraction revealed high relative abundances of *Sphingomonas*, *Tropheryma*, *Acidovorax*, and *Asticcacaulis* in some pneumotype_UN_ samples, but not in pneumotype_SCT_ samples. UniFrac distances between pneumotype_UN_ and saline (0.72 ± 0.02) were lower than the distance between pneumotype_SCT_ and saline (0.78 ± 0.04, *P*= 0.002), also demonstrating a lower signal/noise ratio for samples in pneumotype 1.

### Pneumotype and lung inflammation

**Figure 6:**
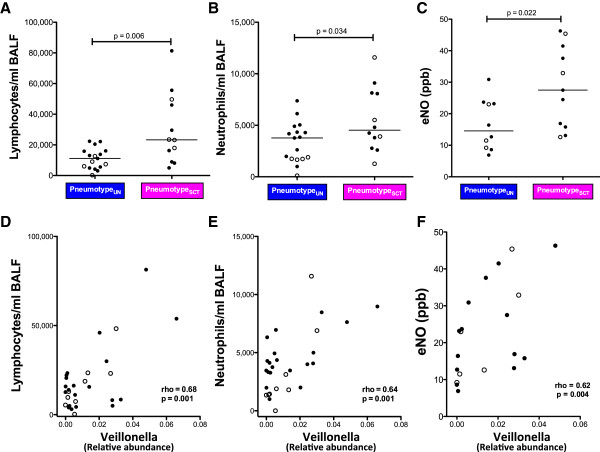
**Relationship between pneumotype/supraglottic-characteristic taxa and lung inflammation.** Inflammatory cells (lymphocytes and neutrophils) and eNO were measured in BAL of asymptomatic subjects (never-smokers in open circles and smokers in closed circles). **(A, B,C)** Comparisons of inflammatory cells and eNO between pneumotype_UN_ and pneumotype_SCT_ (Mann–Whitney). Pneumotype_SCT_ was associated with higher inflammatory BAL cells and higher levels of eNO. **(D, E,F)** Correlations between relative abundance of *Veillonella*, supraglottic-characteristic taxa, in BAL in relation to inflammatory cells/eNO (Spearman rho correlation). *Veillonella* showed a positive correlation with both BAL inflammatory cells and eNO.

Since lymphocytes and neutrophils are recruited into the alveolar space during inflammation, we used BAL cell counts as measures of lung inflammation. Compared with pneumotype_UN_, pneumotype_SCT_ was associated with higher numbers of lymphocytes (11,130 (4,640-16,069)*vs*. 23,315 (10,256-47,706) cells/mL for pneumotype_UN_ and pneumotype_SCT_ respectively, *P*= 0.007) and neutrophils in BAL (3,333 (1,411-4,327)*vs*. 4,537 (3,211-8,259) cells/mL, *P*= 0.034, Figure 6A and B). We also evaluated eNO obtained prior to the bronchoscopy as an independent measure of lung inflammation (Figure 6C). Compared with pneumotype_UN_, pneumotype_SCT_ was associated with higher levels of eNO (14.5 (9.0-23.3)*vs*. 27.5 (15.8-41.5) ppb, *P*= 0.022). Since smoking is known to decrease eNO we investigated whether the association between pneumotype_SCT_ and increased eNO was independent of smoking. First, eNO levels in never-smokers and smokers were similar (17.8 (10.9-36.0)*vs*. 20.0 (13.0-32.6), *P*= 0.71). Further, using a linear regression model that considered eNO as dependent variable and smoking and pneumotype as dichotomized predictors, we found that pneumotype_SCT_ was independently associated with eNO (B = 12.3 ppb, *P*= 0.028) while smoking was not significantly associated with levels of eNO (B = 0.5 ppb, *P*= 0.9). We then evaluated whether relative abundances of specific supraglottic-characteristic taxa were associated with lung inflammation. As shown in Figure 6D, E, and F, the relative abundance of *Veillonella* was positively correlated with both BAL inflammatory cells and eNO. Similarly, relative abundance of *Prevotella* correlated with BAL inflammatory cells and eNO (data not shown). These data indicate that a pneumotype defined by supraglottic-characteristic taxa is associated with inflammatory cells in the lung and with a non-invasive marker of airway inflammation.

## Discussion

This paper described distinct microbial communities that defined two pneumotypes in asymptomatic subjects. Pneumotype_UN_ had low bacterial load and a taxonomic distribution similar (although not identical) to the background microbiome found in sterile saline. Pneumotype_SCT_ had a higher 16S rRNA gene load and was enriched with supraglottic taxa such as *Prevotella* and *Veillonella*. Pneumotype_SCT_ subjects had subclinical lung inflammation with increased BAL inflammatory cells and eNO. These data suggest an association between a distinct human microbiome and inflammation in the lung.

Supraglottic secretions have a 50-fold higher 16S rRNA gene concentration than BAL; therefore, carry-over of oropharyngeal secretions to the lower airways is a major challenge for bronchoscopy. Prior studies suggest that the lower airway microbiome have similar microbial composition to the upper airway, raising concerns for carry-over of oropharyngeal secretions to the lower airways [5,6,9,10]. The route used for bronchoscopic approach might have significant impact since the microbiome of the oral and nasal cavity are different [40,41]. We used the nasal route because of evidence that nasal brushes have low 16S rRNA gene concentrations [27]. Our nasal bronchoscopic technique may have reduced contamination with oral secretions, enabling the uncovering of pneumotype_UN_ with low abundance of supraglottic-characteristic OTUs.

Three lines of evidence suggest that pneumotype_SCT_ was not due to carry-over of supraglottic taxa during bronchoscopy. First, had there been carry-over, the microbiome of first BAL would more closely resemble the supraglottic microbiome than the second BAL. In fact, UniFrac distances between supraglottic samples and the first BAL and second BAL were not different. Second, it is likely that the increased lymphocytes and neutrophils in the alveolar space of pneumotype_SCT_ subjects were almost certainly present prior to bronchoscopy since cell recruitment into the alveolar space probably requires more time than the duration of the bronchoscopy procedure. Third, exhaled NO was increased in pneumotype_SCT_ patients before the research bronchoscopy, demonstrating that this inflammatory phenotype existed prior to bronchoscopy.

In our study population, we found two distinct lung microbiomes. Pneumotype_UN_ had low 16S rRNA concentration, little evidence of supraglottic-characteristic OTUs and a taxonomic composition similar to the normal saline used in the research bronchoscopy. These results differ from recently published series in which oral microbiota was present in most of the BAL samples [6,11]. Technical differences between the studies may account for this discrepancy, since we aimed to minimize any potential carry-over with our bronchoscopic technique. The presence of microbial DNA in sterile saline made it difficult to ascertain which bacterial OTUs, if any, were present in the lung of pneumotype_UN_ subjects prior to the bronchoscopy. To reduce background noise, we subtracted saline-characteristic OTUs from the BAL. This revealed the presence of *Sphingomonas*, *Tropheryma*, *Acidovorax*, and *Asticcacaulis* in some pneumotype_UN_ but not in pneumotype_SCT_ samples. Our finding of *Tropheryma* in 2/16 patients in pneumotype_UN_ (relative abundance of 0.02 in the never-smoker and 0.11 in the smoker) is consistent with a recent report describing *Tropheryma* in the lower airways of 12% to 40% of normal subjects [10]. Studies with larger cohorts using low contamination bronchoscopic techniques, deeper sequencing, and bioinformatic source tracking approaches are needed to better describe pneumotype(s) in subjects with low bacterial rRNA gene concentrations [42,43].

Our clustering analysis allowed us to define pneumotype_SCT_*,* which was present in 8/20 smokers (40%) and 4/9 never-smokers (45%). Prior investigations have attributed the observed increased abundance of oral-characteristic taxa in the lower airways to microaspiration or carry-over. This study extends prior investigations by showing that systematic carry-over does not occur and that the increased abundance of supraglottic-characteristic flora in pneumotype_SCT_ was associated with increased BAL neutrophils, lymphocytes, and eNO. Since pneumotype_SCT_ also has high relative abundance of *Prevotella*/*Veillonella* and high inflammatory cells in the lung, there may be a common link between microaspiration of *Prevotella*/*Veillonella* and inflammation. Importantly, the BAL cell differentials were normal in both never-smokers and smokers, suggesting that differences in lung inflammation between pneumotype_UN_ and pneumotype_SCT_ represent subclinical variation.

Data suggest that smoking alters the upper airway microbiome leading to enrichment with *Veillonella*[11,41]. Persistent microorganisms contribute to pulmonary inflammation in current and former smokers with advanced COPD [44]. Similar to recent studies [11], we found no significant difference in α or β diversity between never-smokers and smokers. It is possible that increasing the sequencing depth would reveal smoking or early COPD-specific microbes present at lower relative abundance. It also is possible that investigation of larger cohorts would reveal more substantial microbiome differences between these never-smokers and smokers. However, perturbation of the lung microbiome may occur only in more advanced disease due to progressive microaspiration and impaired bacterial clearance. Alteration of the lung microbiome also may follow treatments for COPD with antibiotics and/or inhaled steroids. The early nature of disease in our smoking cohort or the absence of recent drug treatment may account for the differences between our observations and the prior reports [7,8,21].

The association between specific pneumotype and airway inflammation was evaluated utilizing eNO based on prior observations describing increased eNO in pulmonary infections and following lipopolysaccharide administration [45-47]. Our results extend these observations by demonstrating that elevated eNO may occur in association with a specific pneumotype even in the absence of overt pneumonia. Increased eNO levels were associated with both pneumotype_SCT_ and enrichment with supraglottic taxa such as *Veillonella* suggesting that the presence of subclinical inflammatory changes may occur in response to pathogen-associated molecules. Although smoking may influence eNO levels, in our cohort, the association of increased eNO with pneumotype_SCT_ was independent of smoking history [48,49]. Further investigation will be needed to determine whether a change in pneumotype leads to increase levels of eNO. The enhanced subclinical inflammation observed in pneumotype_SCT_ may warrant investigation into whether this pneumotype is associated with early physiologic markers of airway dysfunction in smokers at risk for COPD.

This study has several limitations. DNA molecular techniques cannot determine bacterial viability. Further, the association between inflammation and supraglottic-characteristic taxa in the lower airways does not imply that *Prevotella* and *Veillonella* in the lower airways cause inflammation. Additionally, the relatively small sample size, and the depth of sequencing may have prevented us from observing subtle but potentially important differences between smokers and never-smokers. Finally, our cross-sectional design does not allow us to measure the temporal stability of the lower airway microbiome. Evaluation of the impact of pneumotype resilience on lung inflammation is important to understand the potential role of the lung microbiome in the development of lung injury.

## Conclusions

We observed that a pneumotype enriched with supraglottic-characteristic bacteria *Prevotella* and *Veillonella* was associated with higher inflammatory markers, consistent with the hypothesis that oral bacteria in the lung produce subclinical pulmonary inflammation. In this relatively healthy cohort, the association between microbiome and lung inflammation exists in asymptomatic subjects, both never-smokers and smokers. In advancing COPD, mucocilliary dysfunction worsens and the risk for microaspiration increases [50,51]. This could introduce high relative abundance of oral flora into the lower airways leading to pneumotype_SCT_ and its associated increased inflammation. In addition, it is plausible that the progressive immune dysfunction of COPD changes the lung microbiome [52,53]. Further understanding of the association between pneumotypes and pulmonary inflammation will be required to relate the above observations to the development of lung diseases and the potential impact of antibiotics.

## Availability of supporting data

The dataset(s) supporting the results of this article available in the dbGaP repository (phs000633.v1.p1).

## Abbreviations

BAL: Bronchoalveolar lavage; COPD: Chronic obstructive pulmonary disease; DLCO: Diffusion capacity; eNO: Exhaled nitric oxide; FDR: False discovery rate; FEV1: Forced expiratory volume in 1 s; FRC: Functional residual capacity; FVC: Forced vital capacity; IOS: Impulse oscillometry; OTUs: Operational taxonomic units; PCoA: Principal coordinate analysis; qPCR: Quantitative PCR; QIIME: Quantitative Insights into Microbial Ecology; RV: Residual volume; TLC: Total lung capacity.

## Competing interests

The authors declare that they have no competing interests.

## Authors’ contributions

Conception and design: LNS, MJB, MDW. Acquisition of data: LNS, RK, KIB, RMG. Analysis and interpretation of data: LNS, AVA, JCC, BW, HC, MJB, MDW. Drafting or revising of article: LNS, AVA, JCC, KIB, RMG, WNR, MJB, MDW. Final approval of the manuscript: LNS, AVA, JCC, MJB, MDW. All authors read and approved the final manuscript.

## Supplementary Material

Additional file 1: Figure S1 Sampling scheme of background, supraglottic and BAL. Thirty-four background samples were obtained from either sterile saline used for BAL (*n* = 6) or saline passed through bronchoscope’s suctioning channel prior to procedure (*n* = 28). All bronchoscopies were performed nasally. In the 15 of the 29 studied subjects where supraglottic samples were obtained, we used a separate bronchoscope (Bronchoscope 1). This bronchoscope was passed without suctioning until vocal cords were visualized, at which time sample was obtained and then scope was withdrawn. The supraglottic sample was obtained by flushing 10 cc of normal saline through this bronchoscope. For all BAL samples, a separate bronchoscope (Bronchoscope 2) was passed without suctioning until wedged, at which time BAL was obtained. In the subset of 15 of the 29 studied subjects, BAL was differentially obtained from lingula and right middle lobe to evaluate for carry-over of supraglotic microorganisms. For rest of analysis, BAL obtained in the lingula and right middle lobe was pooled in all 29 subjects. Click here for file

Additional file 2 Online supplement: Enrichment of lung microbiome with supraglottic taxa is associated with increased pulmonary inflammation. Click here for file

Additional file 3: Figure S2 Evaluation of BAL microbiome compared with background and supraglottic microbiome in Principal Coordinate Analysis (PCoA). PCoA (x axis PC1 = 26.8% *vs*. y axis PC2 = 8.56%) based on weighted UniFrac distances for microbiome of background (black), BAL (never-smokers in green, smokers in blue), and supraglottic area (red). BAL samples had the highest variability as expressed by their distribution along PC1. Some BAL samples overlapped with background microbiome whereas others overlapped with supraglottic microbiome. (**A**) PCoA weighted by relative abundances of *Propionibacterium* (black boxes) showed higher relative abundances for this taxa in BAL samples that overlapped with background samples. (**B**,**C**) PCoA weighted for relative abundances of *Prevotella* and *Veillonella* (black boxes) showed higher relative abundances for these taxa among BAL samples that overlapped with supraglottic samples. Click here for file

Additional file 4: Figure S3 Comparison between the lung microbiome of never-smokers and asymptomatic smokers. (**A**) PCoA based on weighted UniFrac distances for never smokers and smokers. PC1, PC2, and PC3 represent 43.7% of the variability on the data. Data shows complete overlapping of circle of inertia between smokers and never smokers. (**B**) α-diversity, expressed as richness, was similar in never-smokers and smokers. (**C**) β–diversity, based on weighted UniFrac distance for pairwise comparisons, among and between never-smoker and smoker subjects also was not significantly different (mean±SEM) between the groups. Click here for file
