# Correction: Enrichment of lung microbiome with supraglottic taxa is associated with increased pulmonary inflammation

**DOI:** 10.1186/2049-2618-2-21

**Published:** 2014-07-02

**Authors:** Leopoldo N Segal, Alexander V Alekseyenko, Jose C Clemente, Rohan Kulkarni, Benjamin Wu, Zhan Gao, Hao Chen, Kenneth I Berger, Roberta M Goldring, William N Rom, Martin J Blaser, Michael D Weiden

**Affiliations:** 1 André Cournand Pulmonary Research Laboratory, Bellevue Hospital Center/New York University School of Medicine, New York, NY, USA; 2 Division of Pulmonary and Critical Care Medicine, New York University School of Medicine, New York, NY, USA; 3 Department of Medicine, New York University School of Medicine, New York, NY, USA; 4 Center for Health Informatics and Bioinformatics, New York University School of Medicine, New York, NY, USA; 5 Division of Genetics and Genomic Sciences, Mount Sinai School of Medicine, New York, NY, USA

## Correction

After publication of this work [1], we noted that we inadvertently failed to include the complete list of all co-authors. The full list of authors has now been added and the Authors’ Contributions and Competing Interests sections modified accordingly.

## Competing interests

The authors declare that they have no competing interests.

## Authors’ contributions

Conception and design: LNS, MJB, MDW. Acquisition of data: LNS, RK, KIB, RMG, ZG. Analysis and interpretation of data: LNS, AVA, JCC, BW, HC, ZG, MJB, MDW. Drafting or revising of article: LNS, AVA, JCC, KIB, RMG, WNR, MJB, MDW. Final approval of the manuscript: LNS, AVA, JCC, MJB, MDW. All authors read and approved the final manuscript.
