# Supplementary figures and images for: Enrichment of lung microbiome with supraglottic taxa is associated with increased pulmonary inflammation
Source: Microbiome. 2013 Jul 1;1:19. doi: 10.1186/2049-2618-1-19 (PMC3971609; doi:10.1186/2049-2618-1-19)

Figure S1

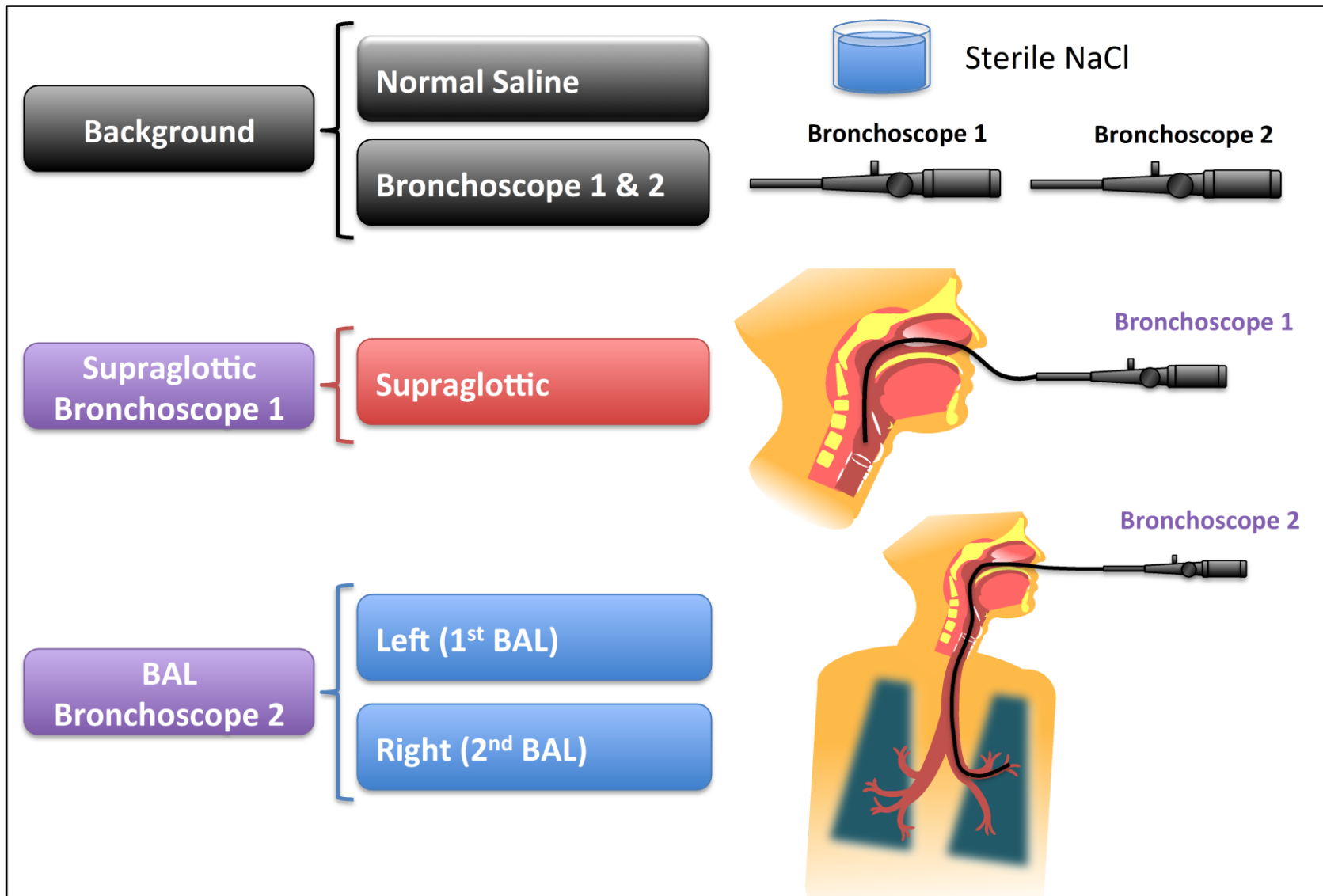

Supplement: Additional file 1: Figure S1 — Sampling scheme of background, supraglottic and BAL. Thirty-four background samples were obtained from either sterile saline used for BAL (n = 6) or saline passed through bronchoscope’s suctioning channel prior to procedure (n = 28). All bronchoscopies were performed nasally. In the 15 of the 29 studied subjects where supraglottic samples were obtained, we used a separate bronchoscope (Bronchoscope 1). This bronchoscope was passed without suctioning until vocal cords were visualized, at which time sample was obtained and then scope was withdrawn. The supraglottic sample was obtained by flushing 10 cc of normal saline through this bronchoscope. For all BAL samples, a separate bronchoscope (Bronchoscope 2) was passed without suctioning until wedged, at which time BAL was obtained. In the subset of 15 of the 29 studied subjects, BAL was differentially obtained from lingula and right middle lobe to evaluate for carry-over of supraglotic microorganisms. For rest of analysis, BAL obtained in the lingula and right middle lobe was pooled in all 29 subjects. [file 2049-2618-1-19-S1.pdf]

Figure S2

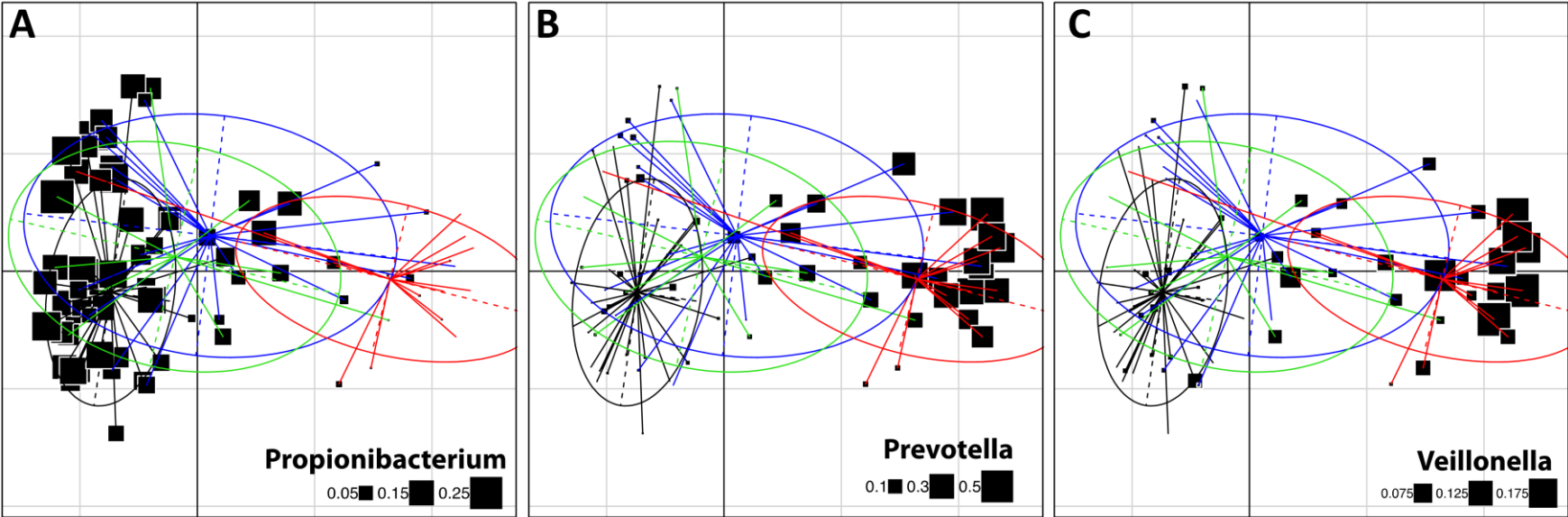

Supplement: Additional file 3: Figure S2 — Evaluation of BAL microbiome compared with background and supraglottic microbiome in Principal Coordinate Analysis (PCoA). PCoA (x axis PC1 = 26.8% vs. y axis PC2 = 8.56%) based on weighted UniFrac distances for microbiome of background (black), BAL (never-smokers in green, smokers in blue), and supraglottic area (red). BAL samples had the highest variability as expressed by their distribution along PC1. Some BAL samples overlapped with background microbiome whereas others overlapped with supraglottic microbiome. (A) PCoA weighted by relative abundances of Propionibacterium (black boxes) showed higher relative abundances for this taxa in BAL samples that overlapped with background samples. (B,C) PCoA weighted for relative abundances of Prevotella and Veillonella (black boxes) showed higher relative abundances for these taxa among BAL samples that overlapped with supraglottic samples. [file 2049-2618-1-19-S3.pdf]

Figure S3

**A**

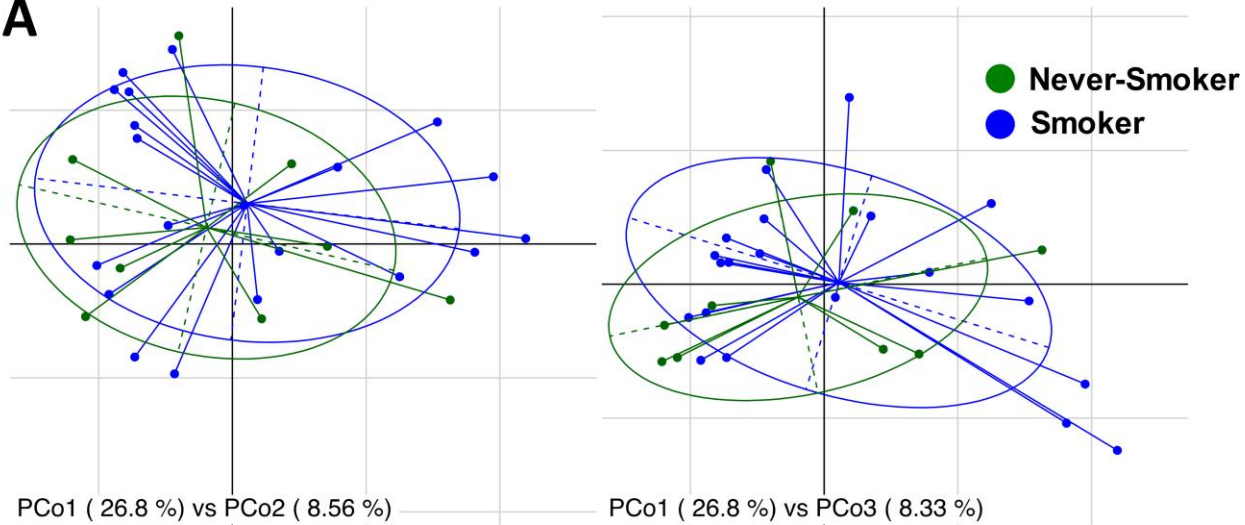

**B**

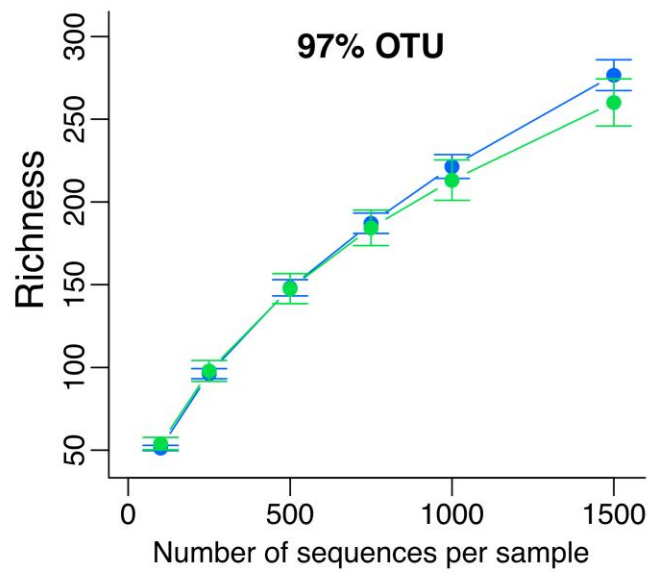

**C**

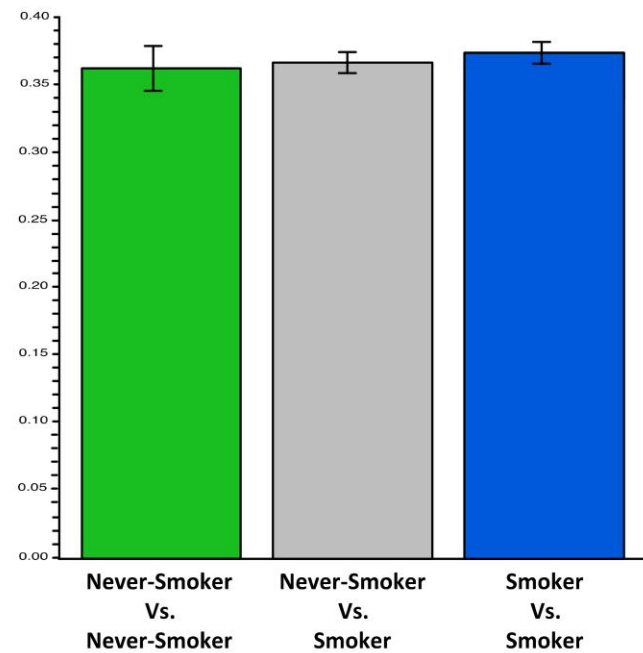

Supplement: Additional file 4: Figure S3 — Comparison between the lung microbiome of never-smokers and asymptomatic smokers. (A) PCoA based on weighted UniFrac distances for never smokers and smokers. PC1, PC2, and PC3 represent 43.7% of the variability on the data. Data shows complete overlapping of circle of inertia between smokers and never smokers. (B) α-diversity, expressed as richness, was similar in never-smokers and smokers. (C) β–diversity, based on weighted UniFrac distance for pairwise comparisons, among and between never-smoker and smoker subjects also was not significantly different (mean±SEM) between the groups. [file 2049-2618-1-19-S4.pdf]
