# Supplementary material for: Enrichment of lung microbiome with supraglottic taxa is associated with increased pulmonary inflammation
Source: Microbiome. 2013 Jul 1;1:19. doi: 10.1186/2049-2618-1-19 (PMC3971609; doi:10.1186/2049-2618-1-19)
Supplement: Additional file 2 — Online supplement: Enrichment of lung microbiome with supraglottic taxa is associated with increased pulmonary inflammation. [file 2049-2618-1-19-S2.pdf]

On line supplement: Enrichment of lung microbiome with supraglottic taxa is associated with increased pulmonary inflammation

Leopoldo N. Segal[1,2,3], Alexander V. Alekseyenko[3,4], Jose C. Clemente[5], Rohan Kulkarni[2], Benjamin Wu[2], Hao Chen[4], Kenneth I. Berger[1,2,3], Roberta M. Goldring[1,2,3], William N Rom[2,3], Martin J. Blaser[3], Michael D. Weiden[2,3]

[1] André Cournand Pulmonary Research Laboratory, Bellevue Hospital Center/New York University School of Medicine, NY

[2] Division of Pulmonary and Critical Care Medicine, New York University School of Medicine, NY

[3] Department of Medicine, New York University School of Medicine, NY

[4] Center for Health Informatics and Bioinformatics, New York University School of Medicine, NY

[5] Division of Genetics and Genomic Sciences, Mount Sinai School of Medicine.

Corresponding Authors:

Leopoldo N. Segal and Michael D. Weiden, MD

Emails: Leopoldo.Segal@nyumc.org; Michael.Weiden@nyumc.org

Methods

Subjects

Non-smokers were enrolled from the NYU CTSI-sponsored Healthy Volunteers Bronchoscopy Cohort (HVBC), characterized by subjects with no significant smoking history, normal spirometry, and absence of pulmonary, cardiovascular, renal, or endocrine disease. Smokers were enrolled from the NYU Early Detection Research Network (EDRN, 5U01CA086137-13) cohort, which consists of approximately 2000 subjects with substantial smoking history (43.8±24.3 pack-years). None of the subjects enrolled had respiratory symptoms or had received any inhaled treatment.

DNA isolation and quantification

Total bacterial and human DNA levels were determined by quantitative PCR (qPCR) using the LightCycler FastStart TaqMan Probe Master (Roche, Germany). Universal primers and probes were used to target conserved regions of the 16S rRNA gene (sequence 5'-3' primers: 8F=AGAGTTTGATYMTGGCTCAG; EUB361R= CGYCCATTGBGBAADATTCC; and probe = 6-FAM-TACGGGAGGCAGCAGT-BHQ1) and hGAPDH (primers: GAP-F=GGGCTCTCCAGAACATCATCC, GAP-R= GTCCACCACTGACACGTTGG; and probe= 6-FAM-CCTCTACTGGCGCTGCCAAGGCT-BHQ1). Duplicate 20 m l reactions were performed under the following reaction conditions: 10 µl Probe Master Mix, 6 µl PCR-grade H2O, 1 µl of forward and reverse primer (10 pM/µl each), 1 µl TaqMan Probe (5 pM/µl) and 1 µl template DNA. Reactions were run on a Roche

Lightcycler 480 Real-Time PCR system (Roche, Germany) with the following cycling conditions: initial denaturing at 95°C for 10 min, followed by 45 cycles of denaturation at 95°C for 10 seconds, annealing at 54°C for 30 seconds, and extension at 72 C for 20 seconds.

PCR amplification for 454 pyrosequencing

Amplicon library preparation was performed with 5 – 20 ng (genomic DNA) using Roche FastStart High Fidelity PCR system, dNT Pack (Roche Applied Science, Germany) and according to manual instructions. The V1–V2 region of the 16S rRNA gene was amplified using a forward primer (27F= CCTATCCCCTGTGTGCCTTGGCAGTCTCAGAGAGTTTGATCCTGGCTCAG) and a barcoded reverse primer (A_338R = CCATCTCATCCCTGCGTGTCTCCGACTCAGA-Barcode-ATGCTGCCTCCCGTAGGAGT). Each reverse primer contained a unique 12-nt barcod [1]. Each unique barcoded amplicon was generated in 50μl reactions with the following reaction conditions: 37.5 μl PCR-grade H2O, 5 μl Buffer with Mg (1.8 mM), 1 μl dNTP (200 mM each), 2 μl of forward and reversed barcoded primer (10 mM), 0.5 μl Taq Blend (2.5 U/reaction) and 2 μl template DNA. Reactions were run on a C1000 Touch Thermal Cycler (Bio-Rad) with the following cycling conditions: initial denaturing at 95°C for 2 min followed by 35 cycles of denaturation at 95°C for 30 seconds, annealing at 55°C for 30 seconds, and extension at 72 C for 30 seconds, with a final extension of 7 min at 72°C. Amplicons were purified (Agencourt AMPure beads, Beckman Coulter) as per the manufacturer instructions, and run through 0.8% agarose gels containing

ethidium bromide to confirm presence of a bright band around the expected region of 350 bp. If the band was not present or faint, the amplification process was re-run. After fluorometric quantification using Quant-iT PicoGreen dsDNA Assay Kit (Invitrogen, UK), amplicons were diluted to 1 x 10^9 molecules/μl and pooled for sequencing using the 454-FLX Titanium.

Upstream informatics analysis of the 16S sequences

Tables of absolute OTU abundances were further using analyzed custom scripts in the R statistical programming environment.[62,63] Bar-coded samples were pooled in sets of 24 and were sequenced in two separate runs. A total of 40 samples were repeated to evaluate reproducibility. The first two pools of 24 samples showed high relative abundance of a unique micrococcus OTU that was not present in repeated runs, suggesting contamination during sampling processing. After upstream removal of this OTU, samples that were repeated in both runs showed no significant differences in relative abundances. Since there was good technical reproducibility between the 454 runs, we merged the data from both runs.

QIIME processing

The following commands were used for processing in QIIME version 1.5.0 (downloaded on 2012/05/10)[2]:

1. Demultiplexing

   split_libraries.py -m <map file for each lane> -f <FASTA file for each lane> -q <Quality file for each lane> -o <output directory>

2. All demultiplexed sequences are merged as a whole FASTA file: suffix_final_id_merged.fna.
3. Pick the OTUs

   pick_otus_through_otu_table.py -i suffix_final_id_merged.fna -o merged_otus
4. Identify possible chimeric OTUs[3]

   identify_chimeric_seqs.py -m ChimeraSlayer -i suffix_final_id_merged.fna -a reference_set_aligned.fasta -o chimeric_seqs.txt
5. View the statistics of the OTU table

   per_library_stats.py -i merged_otus/otu_table.biom
6. Generate the beta diversity

   beta_diversity_through_plots.py -I merged_otus/otu_table.biom -m map_3.txt -o merged_bdiv1099/ -t merged_otus/rep_set.tre -e 1099

Test for supraglottic carry-over

Since contamination of the bronchoscope with supraglottic secretions limits the ability to interpret sequence information derived from the BAL, we evaluated systematic carry-over by comparing 1st and 2nd BAL samples for 15 subjects in whom two bronchoscopes were used. We postulated that supraglottic taxa carry-over would decline between the first and second BAL as the supraglottic secretions contaminating the bronchoscope channel were diluted with 150 ml of normal saline used in each BAL. If supraglottic bacteria were carried into the lower airway by the bronchoscope, we predicted that the 1st BAL would more

closely resemble the supraglottic microbiome than the 2nd BAL. In contrast, we predicted that if the procedure did not produce systematic carryover, the 1st and 2nd BAL would be similarly distant from the supraglottic microbiome.

Results

Bacterial 16S rRNA gene qPCR and sequence.

We quantified the airway total bacterial concentration by performing quantitative PCR for 16S rRNA genes. All 29 subjects had BAL samples and 15 subjects had supraglottic aspirate as well as background samples assayed for bacterial 16S rRNA gene concentration. There was no significant difference in bacterial load between non-smoker (154,687 [59,656-233,041] copies/ml) and smoker groups (62,562 [24,509-133,578] copies/ml, $p = 0.063$). qPCR for human GAPHD showed that there were between 0.5 and 23 copies of bacterial DNA for each copy of human GAPDH in BAL fluid.

Supraglottic and lower airway microbiome

Since the aerodigestive system comprises a series of open conduits capable of transmitting bacteria, we used rRNA gene sequences to define the composition of the supraglottic and lower airway bacterial communities. The microbiome of the sterile saline used to obtain the clinical samples represents background noise added to the analysis by the research bronchoscopy. Interpretation of the lung microbiome, therefore, must take saline background sequences into account.

Further, to assess the contribution of supraglottic and background sequences to the microbial community found in the BAL we performed PCoA analysis combining data from supraglottic and background sequences with BAL data. Supraglottic samples are shown in red and background samples in back. Supraglottic samples and saline sequences were distinct with virtually no overlap in PC1 (**Figure S2,** p = 0.0001). The spatial location of BAL samples varied along PC1 from a space occupied by background samples to a space occupied with supraglottic samples, suggesting two different microbiome populations in the lower airway samples.

To identify OTUs responsible for the differences observed in the BAL microbiome, we calculated the correlation of major taxa with PC1. *Propionibacterium* showed the strongest negative correlation with PC1 (Spearman rho = -0.75) while *Prevotella* and *Veillonella* showed the strongest positive correlation with PC1 (rho= 0.77 and 0.76, respectively, p<0.00001). Since *Propionibacterium* is the most abundant OTUs in sterile saline, its relative abundance represents the contribution of background noise to the BAL microbial community. Since *Prevotella* and *Veillonella* are the most abundant OTUs in the supraglottic samples, we used their relative abundance to represent the enrichment of the lower airway microbiome with supraglottic-characteristic OTUs. To visualize the correlation of *Propionibacterium*, *Prevotella,* and *Veillonella* with PC1, we overlaid the PCoA plot with boxes sized proportionally to the relative abundance of *Propionibacterium*, *Prevotella,* and *Veillonella,* respectively. There is a gradient of *Propionibacterium* in PCoA with box size decreasing from

background to supraglottic samples along PC1 (**Figure S2, Panel A**). BAL samples overlapping with background in PCoA had higher representation of *Propionibacterium*. Conversely, there is a gradient of *Prevotella* and *Veillonella* along PC1 in the opposite direction (**Figure S2, Panel B and C**). BAL samples that overlapped with supraglottic samples had high relative abundance of *Prevotella* and *Veillonella,* similar to what was observed in the microbiome of supraglottic samples.

Effect of smoking on lung microbiome

We then assessed whether the microbial community of the lower airways varied between never-smokers and smokers. There were no significant differences between the microbial communities of these two groups, as assessed by Principal coordinate analysis (PCoA), α diversity, or β diversity. PCoA demonstrated overlap between the microbiome of never-smokers and smokers (**Figure S3, Panel A**). There was no difference in α diversity or β diversity between never-smokers and smokers (**Panel C and D**). Similar results were observed when the smoker group was divided into former and current smokers (data not shown).

References

1. Fierer N, Hamady M, Lauber CL, Knight R: **The influence of sex, handedness, and washing on the diversity of hand surface bacteria.** *Proceedings of the National Academy of Sciences of the United States of America* 2008, **105:**17994-17999.
2. http://qiime.org/home_static/dataFiles.html.
3. http://qiime.org/tutorials/chimera_checking.html.
